# Arrhythmia With Lithium Toxicity Requiring Urgent Dialysis: A Case Report

**DOI:** 10.7759/cureus.21427

**Published:** 2022-01-19

**Authors:** Swarup Sharma Rijal, Ibiyemi Oke, Biraj Shrestha

**Affiliations:** 1 Internal Medicine, Tower Health Medical Group, Wyomissing, USA

**Keywords:** dialysis, lithium poisoning, bradycardia, atrial fibrillation, schizoaffective disorder

## Abstract

Lithium has been widely used as a mood stabilizer. With its narrow therapeutic index, systemic side effects, primarily neurological are a concern. Cardiotoxic effects of lithium are rare, reported as non-specific T-wave flattening, prolonged QT interval, sinus node dysfunction, ventricular tachycardia, cardiomyopathy, and myocardial infarction. We report an interesting case of a young female patient with schizoaffective disorder on lithium who developed life-threatening cardiotoxicity secondary to lithium requiring urgent dialysis.

## Introduction

Lithium is a psychotropic medication with a narrow therapeutic index [1]. At the toxic level, it mostly causes various neurologic abnormalities, but cardiac and renal toxicity has also been reported. Renal toxicity from lithium is almost exclusively seen after at least a decade of therapy. Even then, renal failure occurs in only about 1%. Acute kidney injury following lithium toxicity leads to reduced elimination by the kidneys and this often sets in motion a vicious cycle that requires dialysis for treatment. Reversible atrial fibrillation, A-V block, and intraventricular conduction delay due to lithium toxicity are mostly seen in patients with underlying cardiac disease [2].

## Case presentation

A 37-year-old Caucasian female from a group home presented to emergency with vomiting, poor oral intake, and tremor for one day. Her psychiatric history includes schizoaffective disorder depressed type and intellectual disability, which was being managed chronically with lithium and aripiprazole. Her significant medical history included type 2 diabetes mellitus, essential hypertension, hypothyroidism, and seizure disorder. She was on lamotrigine, lisinopril, levothyroxine, metformin, glipizide, and lithium. The patient had ongoing bilateral upper and lower extremities tremors for the last one week. She started developing multiple episodes of non-bilious vomiting and loss of appetite, subsequently leading to generalized lethargy. She denied any infectious symptoms before this began.

**Figure 1:**
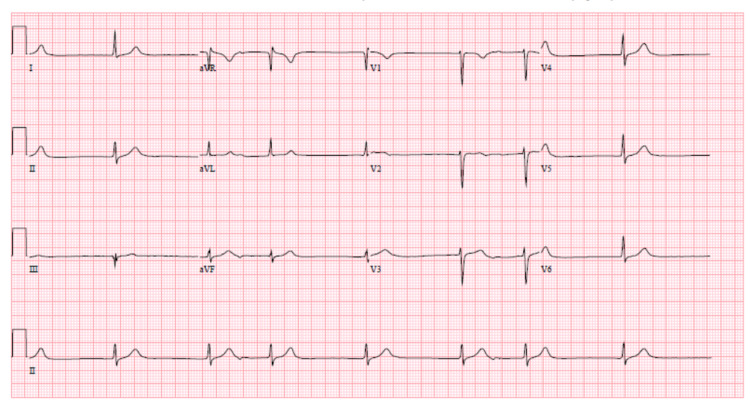
Slow junctional rhythm with the finding of "escape-capture bigeminy" with a prolonged PR interval and the slow ventricular response competing for a junctional pacemaker with a QTc interval of 410 msec

On presentation, she was tremulous, tachycardiac with heart rate 110 beats/minute, blood pressure 100/65 mmHg, afebrile with temperature 37.3 °C, and saturating 95% on room air. Subsequent physical examination was grossly benign. A 12 lead EKG showed a slow junctional rhythm with the finding of "escape-capture bigeminy" with a prolonged PR interval and the slow ventricular response competing for a junctional pacemaker with a QTc interval of 410 msec (Figure 1). Labs revealed normal complete blood count, troponin, thyroid function test, liver function test, and chest radiograph. Her creatinine was 3.24 mg/dL with an estimated GFR of 16.02 (baseline creatinine of 0.74 mg/dL two weeks back). Her potassium was 4.9 mEq/L and sodium 133 mmol/L. The initial lithium level was 2.0 mmol/L (reference value: 0.6-1.2 mmol/L).

**Figure 2:**
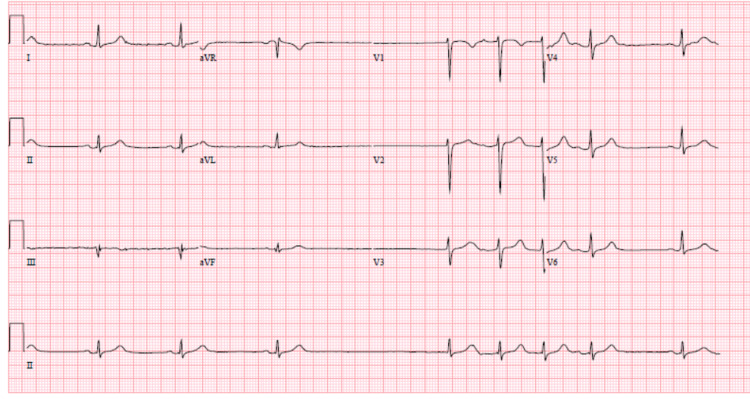
Bradycardia with sinus pauses with heart rate sustaining around 50 beats per minute.

Over the course of four next hours after admission, the patient became more lethargic and the blood serum lithium level rose to 3.7 mmol/L. Initial atrial fibrillation with slow ventricular rate progressed to sinus bradycardia with sinus pauses with heart rate sustaining around 48 beats per minute and transient episodes of spontaneous sinus pauses with heart rate in low 30s (Figure 2). Multiple episodes of sinus pauses were noticed, the longest measuring 3.8 seconds on telemonitoring. With further clinical deterioration, the patient was then transferred to the intensive care unit and emergent dialysis was started. Immediately after 30 minutes of dialysis sinus bradycardia with pauses spontaneously improved to normal sinus rhythm with heart rate around 70 beats per minute with no sinus pauses. Lethargy improved and the repeat lithium level after dialysis was 1.2 mmol/L.

## Discussion

In our case, the appearance of atrial fibrillation with the slow ventricular response, progression to sinus bradycardia with pauses with elevated lithium level, and subsequent recovery after dialysis of a patient with normal lithium level confirmed the relationship between lithium and sinus node dysfunction.

Lithium has been the mainstay for maintenance therapy as a mood stabilizer. The goal of dosing is to achieve a therapeutic level of 0.6-1.2 mmol/L [3]. For the last decade prescription of lithium has been in decreasing trend due to various factors but mainly due to the need for regular monitoring, multi-system side effects [4]. Mild side effects of lithium are usually seen with plasma level >1.5 mmol/L whereas severe side effects are seen with levels >2.5 mmol/L.

Cardiac toxicity has been rarely reported. The prevalence of lithium-induced sinus node dysfunction is not well known [5]. Some of the EKG changes that have been reported include T-wave inversion, sinus bradycardia, sinoatrial blocks, PR prolongation, incomplete bundle branch block, QTc prolongation, increased QT dispersion ratio, Brugada pattern, and ventricular tachyarrhythmias [6]. Lithium competes with sodium, potassium, calcium, and magnesium ions, which plays important role in cellular physiology [7]. Though the exact mechanism of lithium-induced arrhythmias has not been postulated, many hypotheses have been considered. One of the mechanisms is that lithium alters the sinus node pacing function by interacting with pacemaker channels and/or sodium-calcium exchangers [8]. Lithium may also block the pacemaker sodium current, thereby impairing pacemaker activity in the SA node cells [9]. Other intrinsic and/or extrinsic factors also play an important role. These factors include variation in serum level of lithium, degree of cardiac parasympathetic and sympathetic tone, and/or interindividual variation of cardiac sodium current. Also, patients developing sinus node dysfunction may have structural or functional sinus node dysfunction [9].

Treatment modalities for lithium toxicity depend upon the severity of toxicity and the duration of toxicity. Mild side effects resolve after discontinuation of lithium whereas moderate side effects are treated with a combination of fluid with saline diuresis, gastric lavage, and whole bowel irrigation [4]. For severe toxicities like in our case hemodialysis is the treatment of choice [4]. With hemodialysis, there is a concern for the rebound with chronic toxicities as intracellular lithium diffuses slowly from cellular compartments [10,11]. In our case, no rebound was observed after hemodialysis attributing that toxicity was acute.

## Conclusions

Physicians should be aware of uncommon reversible causes of bradyarrhythmia such as lithium toxicity. Although neurological signs and symptoms are often present at the time of presentation, they may be subtle, and diagnosis may require a higher index of suspicion. Renal and cardiac toxicity requires the elimination of the medication from circulation through hemodialysis.
